# A feeder-free, human plasma-derived hydrogel for maintenance of a human embryonic stem cell phenotype *in vitro*

**DOI:** 10.1186/2045-9769-1-6

**Published:** 2012-08-08

**Authors:** Fiona C Lewis, Nicholas Bryan, John A Hunt

**Affiliations:** Clinical Engineering, UKCTE, Institute of Ageing and Chronic Disease, University of Liverpool, Liverpool, L69 3GA UK

**Keywords:** Embryonic stem cells, Hydrogel, Pluripotent stem cells, Cell culture

## Abstract

**Background:**

Human embryonic stem cells (hESCs) represent a tremendous resource for cell therapies and the study of human development; however to maintain their undifferentiated state *in vitro* they routinely require the use of mouse embryonic fibroblast (MEF) feeder-layers and exogenous protein media supplementation.

**Results:**

These well established requirements can be overcome and in this study, it will be demonstrated that phenotypic stability of hESCs can be maintained using a novel, human plasma protein-based hydrogel as an extracellular culture matrix without the use of feeder cell co-culture. hESCs were resuspended in human platelet poor plasma (PPP), which was gelled by the addition of calcium containing DMEM-based hESC culture medium. Phenotypic and genomic expression of the pluripotency markers *OCT4*, *NANOG* and *SOX2* were measured using immunohistochemistry and qRT-PCR respectively. Typical hESC morphology was demonstrated throughout *in vitro* culture and both viability and phenotypic stability were maintained throughout extended culture, up to 25 passages.

**Conclusions:**

PPP-derived hydrogel has demonstrated to be an efficacious alternative to MEF co-culture with its hydrophilicity allowing for this substrate to be delivered via minimally invasive procedures in a liquid phase with polymerization ensuing *in situ*. Together this provides a novel technique for the study of this unique group of stem cells in either 2D or 3D both *in vitro* and *in vivo.*

## Background

Embryonic stem cells isolated from the inner cell mass of pre-implantation embryos are recognised as the most pluripotent of all stem cells and as a result of their distinctive cellular biology have an indefinite capacity to self-renew and give rise to all cell types within the three germ layers of a developing embryo [1][2]. This unique group of stem cells is therefore able to provide a comparable, manipulative platform to model the early stages of human development. When hESCs are cultured directly on tissue culture polystyrene (TCP) their distinct phenotype is quickly lost with cells undergoing spontaneous differentiation characterised by a change in cell morphology and loss of pluripotency markers, such as OCT4 and NANOG [1, 3]. This indicates that their distinctive cell properties are influenced by their microenvironment, thus stringent precisely defined culture procedures have been developed as being fundamentally required to maintain their unique characteristics *in vitro*.

Conventionally, the pluripotency of hESCs has been maintained through co-culture with feeder layers, most commonly MEFs [4], however more recently human feeder cells, such as human fetal muscle [4], human foreskin fibroblasts [5], human uterine endometrial cells and breast parenchymal cells [6] have been used successfully in an attempt to reduce xeno-components. Feeder layers provide both contact signalling and essential exogenous proteins to enable hESCs to expand and self renew, however this approach is labour intensive and often results in inconsistent experimental results, furthermore co-cultivation with MEFs may expose hESCs to potential xeno-contaminants, such as small viruses [7]. While the risk of transmission of murine virus appears to be low, the potential for immune rejection as a result of xeno-proteins does exist, therefore a completely human system is likely to be a prerequisite for any cell-based clinical therapy. In order to make the transition from the laboratory to clinic as simple as possible *in vitro* culture systems should also aim to satisfy this criterion through reducing the use of xeno-components where possible.

A number of cell culture regimes successfully use materials during cell maintenance and expansion. Hydrogels are a particular group of polymeric materials that have significant potential for use with cells as a carrier or ECM as a result of their excellent cellular compatibility. Hydrogels are a class of hydrophilic cross-linked polymers, produced from either organic or inorganic components with a typically >90% water content. This group of materials can therefore provide an extracellular matrix analogue substrate within or on which primary cells can adhere and proliferate [8]. Their hydrophilicity and high water content provides excellent mass transport properties to facilitate influx of soluble factors and efflux of waste molecules, closely resembling the aqueous environment within native soft tissue [9]. Additionally, hydrogels can be delivered via minimally invasive procedures in a liquid phase and then polymerised or gelled *in situ.* The hydrogel engineered for this research utilised the coagulation cascade to achieve gellation, providing cell localisation and stability *in vivo* within 2 minutes post-injection [10].

This unique approach to producing hydrogels for cell culture involves the utilisation of host-derived blood plasma to generate a matrix for cell maintenance and expansion [11]. The coagulation cascade, found natively in blood, was exploited to form a stable organic hydrogel under physiological conditions. Citration of whole blood immediately post-venipuncture chelates free calcium therefore halting coagulation. When the PPP component of the blood is subsequently combined with any culture media containing calcium ions, fibrinogenesis can resume, resulting in a stable hydrogel, formed by the donor’s native clotting system, incorporating the nutrient rich culture media. Furthermore, the exogenous array of growth factors, cytokines and other regulatory peptides found within plasma removes the dependency on xenogenic animal serum from the cell culture matrix.

The PPP-derived hydrogel described herein has previously undergone validation in terms of its ability to maintain the phenotype and expansion of primary dermal fibroblasts, articular chondrocytes and bone marrow derived mesenchymal stem cells for multiple passages *in vitro*[11]. In this study, we demonstrated for the first time, the maintenance of hESCs in an undifferentiated state whilst embedded in a feeder-free, PPP-derived hydrogel construct, retaining both their pluripotent and self- renewal capacity *in vitro*. The simplicity of this culture system along with the opportunities it presents in culturing hESCs in a 3D environment has the potential to advance biological efforts to understand the *in vitro* self renewal of this unique group of cells, whilst meeting the feeder-free demands of today’s research community.

## Results

### *In vitro* validation of PPP-derived hydrogel

A fundamental feature of any culture system is its cytocompatibility therefore hESCs embedded within this hydrogel were subjected to live/dead staining in order to validate if the hydrogel construct was able to provide an environment capable of maintaining sufficient hESC viability. Components of the cytotoxicity kit were found to be freely permeable in the PPP hydrogel. Following treatment with ethanol, hESCs were found to exclusively fluoresce red (not shown) indicating that the hydrogel does not inhibit the diffusion of the assay to the cells embedded within the matrix.

**Figure 1:**
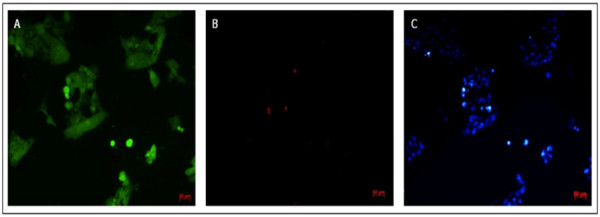
**HESC Viability.** Single field in which live/dead staining of hESCs embedded within PPP-derived hydrogel is shown after 72 hours in culture. **A**: Live cells (green). **B**: Dead cells (red). **C**: Nuclei (blue).

Subsequent viability tests confirmed that hESCs cultured within the PPP-derived hydrogel remained viable (green fluorescence) over 72 hours *in vitro*. Although occasionally cells fluorescing red were identified, indicating the binding of ethidium bromide to the DNA of cells with damaged membranes, this was rare and did not indicate any unusual loss of cell viability (figure 1).

### HESC morphology following culture in PPP-derived hydrogel

**Figure 2:**
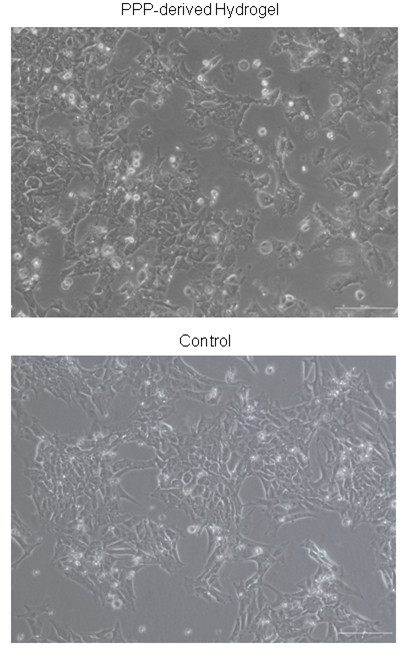
**Cellular Morphology.** Light microscopic observation of confluent hESCs after 72 hours maintained in PPP-derived hydrogel and on fibronectin. Scale bar represents 200 μm.

Undifferentiated hESCs typically exhibit a high nucleus to cytoplasm ratio and form colonies. hESCs embedded within the hydrogel and cultured using DMEM/F12 as detailed previously were observed to proliferate effectively *in vitro* and had successfully formed colonies displaying typical hESC morphology 24 hours following their seeding within the hydrogel. Expansion of the hESC populations continued until reaching confluency after 72 hours as illustrated in figure 2.

### Identification of pluripotency markers

**Figure 3:**
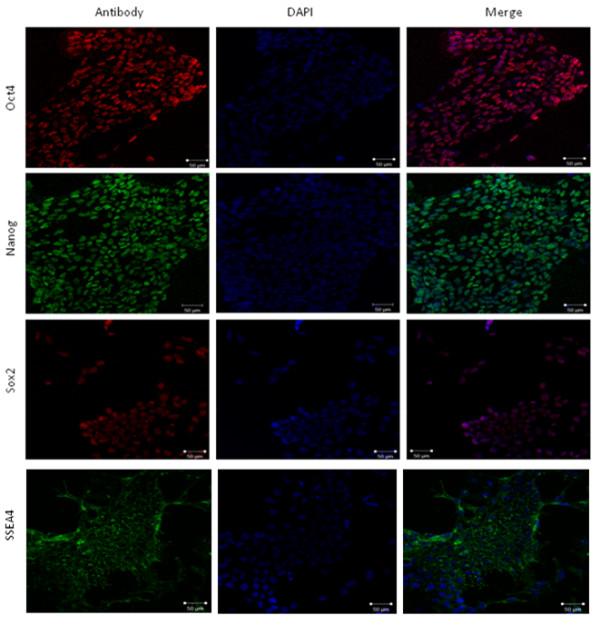
**Identification of Pluripotency Markers.** Fluorescent observation of passage 25 hESCs immunohistochemically stained for OCT4, NANOG, SOX2 (left), DAPI (centre), merged (right) under PPP-derived hydrogel conditions. Scale bar represents 50 μm.

Morphological analysis of hESCs embedded within the hydrogel suggested that hESCs remained undifferentiated. To confirm this observation, immunohistochemistry was performed on the seeded PPP-derived hydrogels to identify pluripotency markers characteristic of hESC undifferentiated phenotype: the transcription factors *OCT4* *NANOG* and *SOX2*, all of which are typically expressed by undifferentiated hESCs in standard culture [12–14] along with the surface antigen SSEA4 were highlighted as indicators of a pluripotent phenotype. All nuclear transcription factors were clearly identified up to 25 passages as illustrated in figure 3.

Expression of the pluripotency markers *OCT4*, *NANOG* and *SOX2*, were also analysed at the gene level by performing qRT-PCR. Both hESCs cultured under PPP-derived hydrogel and fibronectin culture conditions up to 10 passages were analysed. Expression of each of these markers was identified in all samples.

**Figure 4:**
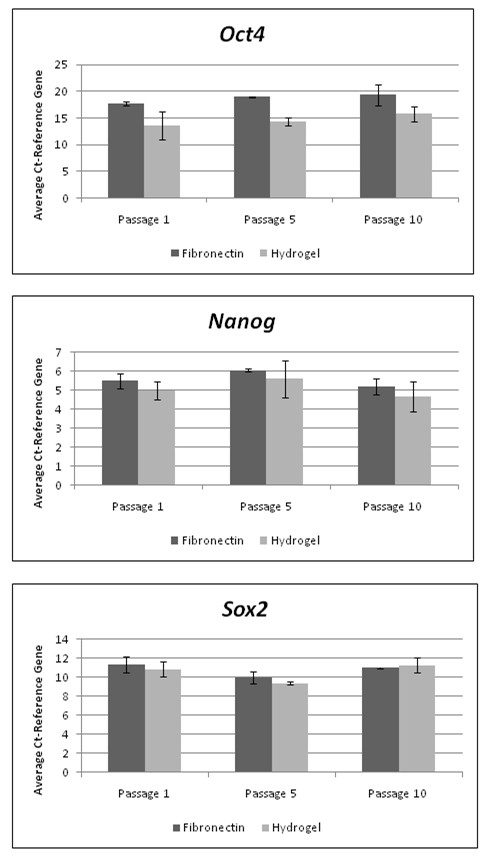
**Pluripotency Gene Expression.** Quantification of pluripotency gene expression after culture under PPP-derived hydrogel and fibronectin culture conditions at passage 1, 5 and 10. Error bars represent 1 standard deviation from the mean, n = 3.

Over the period of culture, expression of OCT4, *NANOG* and *SOX2* was comparable between both hESCs maintained using both PPP-hydrogel and fibronectin conditions over a number of passages with no significant difference in transcript expression (figure 4).

### Maintenance of hESC phenotype

**Figure 5:**
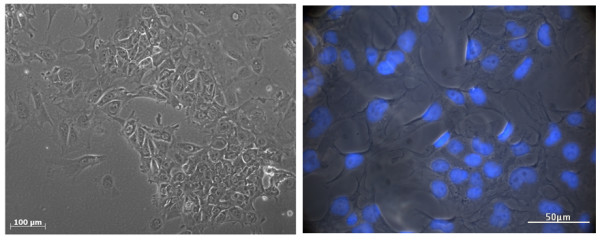
**Maintenance of hESC Phenotype.** Light microscopic observation of hESC morphology after 25 passages maintained under PPP-derived hydrogel conditions, nuclei stained with DAPI (blue).

HESCs maintained within the PPP-hydrogel were cultured through 25 passages with no change in morphology, viability or phenotype observed over the extended period of culture (figure 5).

**Figure 6:**
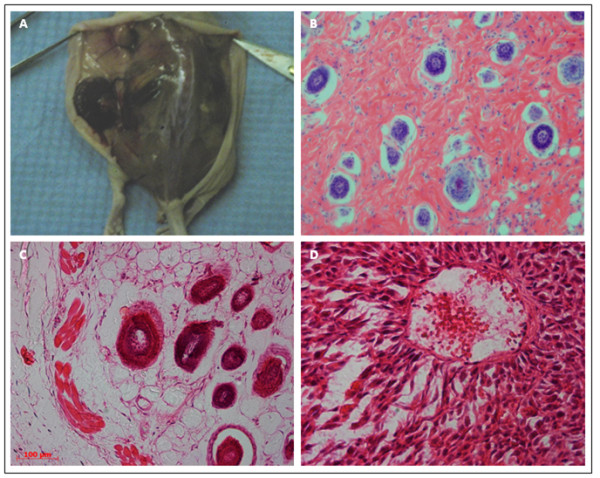
**Teratoma Formation.** Teratoma formation in immunodeficient mice demonstrates differentiation into derivatives of the three germ layers. **A**: Macroscopic observation of teratoma formation. **B**: Glandular structures (endoderm). **C**: Muscle (mesoderm), adipose (mesoderm), hair follicles (ectoderm). **D**: Blood vessels (mesoderm). Scale bar represents 100 μm.

Implantation of hESCs maintained up to passage 25 led to the formation of teratomas 6 weeks post-injection observed both macroscopically and microscopically in each case, n = 6. Derivatives of all three germ layers were identified including endoderm-derived glandular structures, ectoderm –derived hair follicles and a variety of mesoderm-derived structures including adipocytes, myotubes and blood vessels. This provided further confirmation that hESCs maintain their differentiation potential when maintained using this novel PPP-derived hydrogel culture system (figure 6).

## Discussion

Due to their distinctive biology, hESCs represent a promising source of cells for modelling early development and specific diseases whilst potentially providing a source of replacement cells for a number of degenerative diseases. However their culture, expansion and differentiation requires further advancement in order to start to reliably and safely realise the promise of this unique group of stem cells.

In an attempt to provide the optimum environment for hESCs *in vivo,* the properties of the natural 3D microenvironment should be considered as this provides both a structural framework and morphogenic cues mediating cell fate and function; natural extracellular matrices (ECMs) are largely composed of protein fibres distributed within a hydrated network of glycosaminoglycan chains [15] within this matrix are insoluble molecules, such as fibronectin and laminin. In addition there are soluble macromolecules, such as growth factors and cytokines, which are potent stimulators driving cellular functions, such as cell proliferation and differentiation [16–18].

The PPP-derived hydrogel possesses many ideal characteristics which give it excellent cytocompatibility; such as its 3D composition, mechanical integrity and mass transport properties. In terms of its ability to maintain hESCs, the incorporation of fibronectin within the hydrogel construct may serve as an important mediator. As previous studies have shown fibronectin plays an important role in embryonic development where it provides a substratum for guiding gastrulation and the migration of neural crest cells [19]. While fibronectin is one of the best studied non-collagenous ECM proteins it is also moderately abundant in human blood plasma where it contributes to blood clotting and wound healing [20] therefore it is likely that fibronectin within this PPP-derived hydrogel permits the attachment of hESCs through α5β1 integrin binding to RGD sites distributed along fibronectin chains [21, 22]. Both fibrinogen and fibrin also have multiple interacting sites serving as adhesion motifs, including binding sites for α5β1 integrins [22], it is therefore also possible that the abundance of fibrin within the hydrogel may also serve to mediate successful attachment and proliferation of hESCs. In addition the conversion of the soluble protein fibrinogen to the insoluble protein fibrin during gellation generates a network of fibres analogous to that found within natural ECM and in combination with the soluble growth factors supplied in the culture media may serve to generate a microenvironment which effectively mimics the histoarchitecture encountered *in vivo* thus promoting a self-renewing, undifferentiated phenotype.

This investigation has demonstrated, for the first time, the maintenance of hESCs in an undifferentiated state using a feeder-free, PPP-derived hydrogel. This culture system retained both the pluripotency and self renewal capacity of hESCs *in vitro* over long- term culture >25 passages, providing a novel system for the study of this unique group of stem cells *in vitro* in 2 and 3 dimensions. The components of this system are relatively inexpensive and straightforward to use, overcoming many of the barriers currently hindering hESC research and making this system an attractive alternative to currently available feeder-free systems. Validation of this system was undertaken using the well characterised HUES7 cell line, with no detrimental effects on their long- term phenotypic stability observed. This opens up the possibility of successfully using this system for the maintenance of a wide variety of ESC lines.

Due to the incorporation of N2 and B27 supplements in the culture media this system is not xeno-free therefore would not be considered suitable for clinical application at present however further refinements would open up the possibility of direct implantation of stem cell populated hydrogels. The PPP-derived hydrogel has previously been investigated in terms of immunogenicity and minimal host immune cell recruitment upon injection in immunocompromised mice was observed. Furthermore implantation of hESCs embedded within this hydrogel may experience reduced immune responses as a result of their segregation within this construct.

With a view to the future provision of autologous pluripotent cells, such as iPSCs, the culture system would be ideal in many ways. Wide scale application for a broad array of cell therapies may be established with the hydrogel largely generated using a patient’s own plasma. While research into iPSCs is still in its infancy it is likely that safer ways of reprogramming somatic cells will be developed and will thus require the same optimisation of cell culture and maintenance as hESCs. Therefore a feeder-free, potentially-autologous system such as this would provide a platform for clinical translation of iPSCs.

## Methods

### Ethics statement

All research involving human participants was approved by the Institutional Review Board of the University of Liverpool. Following thorough discussion of the intent and risks associated with the study, subjects gave written informed consent to participate in this study.

### Cell culture

The hESC line (HUES7) was obtained from Harvard University (hES Cell Facility/Melton Laboratory, MA, USA). Initial populations were expanded on MEFs then subsequently seeded in feeder-free conditions on TCP dishes pre-coated for 1 hour with 50 μg/ml of human plasma fibronectin (Millipore, UK) solution at 37°C. The media used to maintain hESCs in all cases was composed of DMEM/F12 (Lonza, UK) basal medium supplemented with 10 ng/ml Activin A (R&D, UK), 4 ng/ml NT4 (Preprotech, UK), 100 ng/ml bFGF (Autogen Bioclear, UK), N-2 supplement, B-27 supplement, 1 mM L-glutamine, 1% NEAA, 0.1 mM beta-mercaptoethanol (Invitrogen, UK), 0.1% bovine serum albumin (BSA) (Sigma, UK).

### PPP isolation

20 mL of peripheral blood was isolated from 5 healthy donors of both genders under informed consent via venipuncture and collected in sterile falcon tubes. Immediately post-collection coagulation was prevented by citration using 10% (v/v) 10 mM sodium citrate (Sigma Aldrich, UK). Citrated blood samples were allowed to remain at room temperature for 2 hours and then incubated at 42°C overnight. After 12 hours the blood cells and plasma had largely separated. Samples were centrifuged at 15 g for 10 minutes and the resulting supernatant decanted into clean falcon tubes. The supernatant was centrifuged at 15 g for 10 minutes and once again decanted, taking care not to disturb the cell pellet. Finally 0.22 μm filtration was performed to ensure complete removal of residual erythrocytes and maximal depletion of platelets. The citrated PPP was then pooled and stored at −20°C.

### PPP-derived hydrogel cell culture

The PPP-derived hydrogel was prepared from two distinct components, citrated PPP and hESC media. Both components were pre-warmed to 37°C and the PPP fraction added to the media at a concentration of 10% (v/v), and quickly mixed once to ensure a homogenous amalgamation. The combined solution was incubated for 5–10 minutes at 37°C, in a humidified atmosphere containing 5% CO_2_, allowing the formation of a stable hydrogel. In order to capture undifferentiated hESCs within the hydrogel construct, citrated PPP was combined with a hESC suspension containing 2.5 x 10^5^ cells/mL at a concentration of 10% (v/v). This hydrogel/cell suspension was added to individual wells of a 24 well plate at a volume of 1 mL/well and upon gellation at 37°C, 5% CO_2_ a stable hydrogel was formed with hESCs dispersed throughout. The hydrogel/hESC gels were cultured in DMEM/F12 as detailed previously at 37°C, 5% CO_2_.

After reaching 70% confluency (approx 72 hours), hESCs were passaged with 1x trypsin/EDTA (Invitrogen, UK) for less than 1 minute at 37°C. The trypsinisation reaction was then neutralised with an equal amount of trypsin inhibitor (Invitrogen, UK) and this cell suspension was centrifuged at 400 g for 3 minutes at room temperature. Once the resulting supernatant had been aspirated the cell pellet was resuspended in hESC media and once again encapsulated within a PPP-hydrogel. hESCs were successfully cultured up to passage 25.

### Culture on fibronectin

A human fibronectin culture system, which has previously been validated in terms of its ability to maintain the phenotype of hESCs over multiple passages *in vitro*[23], was used to compare the novel hydrogel with an established feeder-free culture system. This human fibronectin system facilitates the undifferentiated expansion of hESCs via β1-integrin attachment in DMEM/F12 containing FGF2, activin A, neurotrophin 4, N2 and B27 supplements.

Plates were coated with 50 μg/mL human plasma fibronectin solution and incubated for 1 hour at 37°C. This solution was aspirated and hESCs were seeded at a density of 7.5 x 10^5^/mL. hESCs were maintained in DMEM/F12 as detailed previously at 37°C, 5% CO_2_.

### Cell viability

The viability of hESCs cultured using this hydrogel system was evaluated using Live/Dead Viability/Cytotoxicity Kit (Molecular Probes, Invitrogen, UK). The individual components of the kit, consisting of Calcein AM and ethidium homodimer-1 (Ethd-1), were diluted in PBS to give final working concentrations of 2 μM calcein AM and 4 μM EthD-1. Nuclei were counter stained using Hoechst. One hundred μl of each pre-diluted solution were added to each well containing hydrogel-cell matrix and incubated at 37°C for 30 minutes. An inverted fluorescent microscope (Axiovert, Zeiss, UK) was used to visualise hESCs. In order to confirm that the components of this assay were able to diffuse throughout the hydrogel, so as to successfully interact with the cells trapped within it, a control was established whereby hESCs embedded within the hydrogel were subjected to 70% ethanol for 10 minutes, effectively inducing cell death.

### Immunohistochemistry

All samples were fixed *in situ* with 4% (v/v) paraformaldehyde (Sigma Aldrich, UK) for 10 minutes at room temperature. Samples were then blocked in a solution of PBS with 10% serum (Invitrogen, UK) and 0.1% Triton-X100 (Sigma Aldrich, UK) for 1 hour at room temperature. Serum was specific to the animal in which the secondary antibody was raised. Samples were then incubated with a primary antibody or relevant isotype control for 2 hours at 37°C diluted in animal serum at a concentration of 1% in PBS with Triton-X100 at a concentration of 0.1%. The working concentrations of the primary antibodies used were as follows: OCT4 clone 10 monoclonal mouse anti human IgG2b 1:500 (Santa Cruz), NANOG polyclonal goat anti human IgG 1:200 (Cat. No. AF1997), SOX2 clone 245610 monoclonal mouse anti human IgG2a 1:100 (R&D Systems, UK). Upon removing the primary antibody solution, hydrogel embedded samples were washed in PBS three times for 5 minutes at room temperature and the relevant secondary antibody: Alexafluor 594 goat anti mouse IgG2b, Alexafluor 488 rabbit anti goat IgG and Alexafluor 594 goat anti mouse IgG2a (Invitrogen, UK) applied for 1 hour at 37°C. In order to remove any residual secondary antibody, samples were washed three times for 5 minutes at room temperature then mounted onto a microscope slide. To establish positive controls, hESCs cultured using the fibronectin system were stained using the same antibodies. In all samples cell nuclei were counterstained with vector shield mounting medium containing the fluorescent stain 4’,6’-diamidino-2-phenylindole (DAPI) (Vector Laboratories, UK) and each sample was then observed immediately using laser- scanning confocal microscopy (Carl Zeiss LSM 510).

### Real-time PCR (qRT-PCR)

Total cellular RNA was isolated from hESCs cultured under both hydrogel and fibronectin conditions using an RNeasy Mini Kit (Qiagen, UK) according to the manufacturer’s recommendations and eluted in 30 μl of RNase free water. The quantity and purity of RNA was determined by 260/280 nm absorbance using a spectrophotometer. For cDNA synthesis 2 μl of total RNA was used as a template and reverse transcription was performed using SuperScript^TM^ III and OligodT primers (Invitrogen, UK). For subsequent RT-PCR reactions cDNA synthesised from the reverse transcription reaction was diluted 10 fold with ultraPURE DNase and RNase free ddH_2_O (Invitrogen, UK).

All primers were designed in house to amplify the following pluripotency associated genes: *OCT4*, *NANOG*, and *SOX2* while the housekeeping gene β-actin was also amplified to act as a reference. Primers are detailed in Table 1. After optimisation of the primers to ensure their efficiency and ability to amplify a single PCR product, qRT-PCR was performed on all samples including negative controls using a thermocycler (Bio-Rad, UK) and a stock solution containing SYBR Green single tube real time master mix (Bio-Rad, UK), ultraPURE ddH_2_O and a primer solution containing both sense and anti-sense custom designed primers (Invitrogen, UK). Briefly, samples were denatured for 3 minutes at 95°C then cycled 40 times at 95°C for 30 seconds, followed by 40 cycles at the primer specific annealing temperature ranging from 53°C to 57°C, 55°C for 30 seconds, 95°C for 30 seconds and finally 40 cycles at 55°C for 10 seconds. Triplicate readings were taken for each experimental sample and normalised against the reference gene β-actin. The fold change of each target gene was then calculated using 2 ^–(ΔCT sample –ΔCT control)^ were ΔC_T_ is (C_T_ gene of interest-C_T_ reference gene).

**Table 1:** **Primer sequences used in qRT-PCR**

Target gene	Accession No.	Sense Primer	Antisense Primer	Tm (°C)
*OCT4*	NM002701.4	TGGCTCTGCTGACACATC	TGGTTCGCTTTCTCTTTCG	53.4
*SOX2*	NM003106.2	GAGAGAAAGAAAGGGAGAGAAG	GAGAGAGGCAAACTGGAATC	53.0
*NANOG*	NM024865.2	CTGGCTGAATCCTTCCTCTCC	TGATTAGGCTCCAACCATACTCC	57.0
*Β-Actin*	NM001101	GGACCTGACTGACTACCTC	GCCATCTCTTGCTCGAAG	53.9

### Teratoma formation assay

hESCs were maintained within PPP-derived hydrogels to passage 10, harvested using trypsin then resuspended in an appropriate volume of hESC media. Pre-warmed PPP was added at a volume of 10% just prior to implantation and gellation ensued *in situ* under physiological conditions. Six week old, male immunodeficient CH1 mice received four injections into subcutaneous tissue above each shoulder and hip joint with each site receiving 10x^6^ hESCs contained within 300μL PPP-derived hydrogel solution. Animals were sacrificed at 4, 6 and 8 weeks post-implantation. All *in vivo* experimental procedures were conducted in accordance with UK Home Office regulations under licence and were approved by Research Ethics Committee. Tumours were fixed in periodate-lysine-paraformaldehyde fixative for 7 days at 4°C then into cold washing solution for 24 hours at 4°C. Cold acetone was applied to dehydrate samples for a further 5 days at 4°C before replacing with cold Technovit infiltration solution (Heraeus Kulzer) for 1 week at 4°C. This was then replaced with cold Technovit resin embedding solution (Heraeus Kulzer) and the resin surface covered with mineral oil (Sigma Aldrich, UK) for 5 days at −55°C. The temperature was then slowly increased first to −20°C for 2 days then brought to room temperature to allow polymerisation of the tissue blocks. Sections were cut to 6 μM using a polycut microtome and placed onto APTES coated slides. Histological analysis was performed using hematoxylin/eosin staining and observed using transmitted light microscopy.

### Statistical analysis

Statistical analysis was performed using SPSS (SYSTAT Software Inc.) to determine differences in gene expression between the two systems. Samples were evaluated by performing a Mann–Whitney *U* test with a *p*-value of <0.05 being considered statistically significant. In each experiment triplicate qRT-PCR reactions were performed for each individual sample and the average of these values taken to reduce experimental error. The results presented here were obtained from three separate experiments, *n* = 3 for each time point.
